# 胸腔镜治疗肺部微小结节（129例报告）

**DOI:** 10.3779/j.issn.1009-3419.2017.01.05

**Published:** 2017-01-20

**Authors:** 通 王, 天生 闫, 峰 万, 少华 马, 可毅 王, 京弟 王, 金涛 宋, 未 贺, 洁 白, 亮 金

**Affiliations:** 100191 北京，北京大学第三医院胸外科 Department of Thoracic Surgery, Peking University Third Hospital, Beijing 100191, China

**Keywords:** 肺肿瘤, 电视胸腔镜手术, 肺磨玻璃样结节, Lung neoplasms, Video-assisted thoracic surgery, Ground glass opacity

## Abstract

**背景与目的:**

影像技术的发展导致肺部微小结节尤其是肺磨玻璃结节（ground-glass opacity, GGO）检出逐年增多，但术前定性困难。本研究探讨肺部微小结节的临床诊断及微创手术治疗的必要性和可行性、病理诊断，微创切除及淋巴结切除的手术方式。

**方法:**

对2013年12月-2016年11月接受电视胸腔镜手术（video-assisted thoracic surgery, VATS）治疗并有明确病理诊断的共129例患者的临床资料回顾性分析。所有患者术前行薄层计算机断层扫描（computed tomography, CT）扫描，其中21个微小结节术前行CT引导下Hook-wire定位，并根据病理性质及患者身体状况采用不同手术方式。

**结果:**

共129个微小结节，实性结节（solid pulmonary nodule, SPN）37个，恶性比例是24.3%（9/37），术后病理结果为：肺原发性鳞状细胞癌3个，浸润性腺癌（invasive adenocarcioma, IA）3个，转移癌2个，小细胞肺癌（small cell lung cancer, SCLC）1个，错构瘤16个，其他炎症等良性病变12个；49个混合性GGO（mixed ground-glass opacity, mGGO）的恶性比例是63.3%（31/49），术后病理结果为：IA 19个，微浸润腺癌（micro invasive adenocarcioma, MIA）6个，原位腺癌（adenocarcioma *in situ*, AIS）4个，非典型性腺瘤样增生（atipical adenomatous hyperplasia, AAH）1个，SCLC 1个，炎症等良性病变18个；43个纯GGO（pure ground-glass opacity, pGGO）的恶性比例是86.0%（37/43），术后病理结果为：AIS 19个，MIA 6个，IA 6个，AAH 6个，炎症等良性病变6个；GGO总的恶性比例是73.9%（68/92）。52个良性病变均采用VATS肺楔形切除；原发性非小细胞肺癌（non-small cell lung cancer, NSCLC）共73例，VATS肺叶切除和淋巴结清扫33例，VATS肺楔形切除和选择性淋巴结切除6例，VATS肺段切除和选择性淋巴结切除6例，VATS肺楔形切除28例；2个转移癌和2个SCLC，采用VATS肺楔形切除术。另有6例患者术中冰冻病理存在误差，其中2例选择二次手术行肺叶切除和淋巴结清扫。45例有淋巴结病理结果NSCLC只有两例以SPN为表现的IA出现纵隔淋巴结转移，其余均未出现淋巴结转移。术后随访1个月-35个月，平均（15.1±10.2）个月，无复发及转移。

**结论:**

肺部微小结节尤其是GGO，是恶性病灶的概率大，应积极外科处理；围手术期应与患者及家属充分告知冰冻病理结果存在误差可能性，避免医疗纠纷。

## Abstract (en)

**Background and objective:**

The development of image technology has led to increasing detection of pulmonary small nodules year by year, but the determination of their nature before operation is difficult. This clinical study aimed to investigate the necessity and feasibility of surgical resection of pulmonary small nodules through a minimally invasive approach and the operational manner of non-small cell lung cancer (NSCLC).

**Methods:**

The clinical data of 129 cases with pulmonary small nodule of 10 mm or less in diameter were retrospectively analyzed in our hospital from December 2013 to November 2016. Thin-section computed tomography (CT) was performed on all cases with 129 pulmonary small nodules. CT-guided hook-wire precise localization was performed on 21 cases. Lobectomy, wedge resection, and segmentectomy with lymph node dissection might be performed in patients according to physical condition.

**Results:**

Results of the pathological examination of 37 solid pulmonary nodules (SPNs) revealed 3 primary squamous cell lung cancers, 3 invasive adenocarcinomas (IAs), 2 metastatic cancers, 2 small cell lung cancers (SCLCs), 16 hamartomas, and 12 nonspecific chronic inflammations. The results of pathological examination of 49 mixed ground glass opacities revealed 19 IAs, 6 micro invasive adenocarcinomas (MIAs), 4 adenocarcinomas *in situ* (AIS), 1 atypical adenomatous hyperplasia (AAH), 1 SCLC, and 18 nonspecific chronic inflammations. The results of pathological examination of 43 pure ground glass opacities revealed 19 AIS, 6 MIAs, 6 IA, 6 AAHs, and 6 nonspecific chronic inflammations. Wedge resection under video-assisted thoracoscopic surgery (VATS) was performed in patients with 52 benign pulmonary small nodules. Lobectomy and systematic lymph node dissection under VATS were performed in 33 patients with NSCLC. Segmentectomy with selective lymph node dissection, wedge resection, and selective lymph node dissection under VATS were performed in six patients with NSCLC. Two patients received secondary lobectomy and systematic lymph node dissection under VATS because of intraoperative frozen pathologic error that happened in six cases. Two cases of N2 lymph node metastasis were found in patients with SPN of IA.

**Conclusion:**

Positive surgical treatment should be taken on patients with persistent pulmonary small nodules, especially ground glass opacity, because they have a high rate of malignant lesions. During the perioperative period, surgeons should fully inform the patients and family members that error exist in frozen pathologic results to avoid medical disputes.

随着影像技术的发展，直径≤10 mm的肺部微小结节^[1]^的检出逐年增多^[2]^，但术前定性困难，尤其是以磨玻璃结节（ground-glass opacity, GGO）为表现的肺腺癌，肺部微小结节的诊治已成为胸外科热点问题^[3]^。肺部微小结节尤其是微小GGO的诊断与治疗一直是一个难题，采用定期影像学复查，不仅增加了患者的心理负担^[4]^，更有可能延误诊治，导致肿瘤进展。2013年12月-2016年11月对129个肺部微小结节进行胸腔镜手术，取得较好的临床效果，报道如下。

## 1 资料与方法

### 1.1 临床资料

2013年12月-2016年11月，共有129例单发直径≤10 mm肺部微小结节患者在我院接受手术治疗并有明确病理诊断。男性55例，女性74例，年龄22岁-80岁，平均（54.5±13.9）岁，右肺81个，左肺48个。直径4 mm-10 mm，平均（8.1±1.9）mm，SPN 37个，GGO 92个，其中混合性GGO（mixed GGO, mGGO）49个，纯GGO（pure GGO, pGGO）43个。其中7例患者有肺外恶性肿瘤史，其余患者均体检发现，无恶性肿瘤病史。

手术选择标准：随访过程中结节增大、密度增高、实性成分增多等恶性影像表现；或者经抗炎治疗后无明显变化或持续存在；影像学提示恶性程度高（如分叶、毛刺、血管集束征等）；或者有肺癌危险因素，如高龄、家族史或者恶性肿瘤病史、吸烟史等；或者患者心理焦虑严重，影像生活工作及学习，要求手术治疗。

### 1.2 手术方法

采用静脉复合麻醉下双腔气管插管，健侧卧位手术。电视胸腔镜手术（video-assisted thoracic surgery, VATS）手术时时全面检查胸腔，并伸入手指探查并结合影像或根据Hook-wire定位判断结节的具体位置，距离结节2 cm以直线型切割缝合器楔形切除病灶，送快速冰冻切片检查，根据病理结果及患者的心肺功能状态决定下一步手术方案，如VATS肺叶切除术加系统淋巴结清扫；肺段切除加选择性淋巴结切除；肺楔形切除术和（或）选择性淋巴结切除等。

## 2 结果

### 2.1 手术结果

52个良性病变均采用VATS肺楔形切除；原发性非小细胞肺癌（non-small cell lung cancer, NSCLC）共73例，VATS肺叶切除和淋巴结清扫33例，VATS肺楔形切除和选择性淋巴结切除6例，VATS肺段切除和选择性淋巴结切除6例，VATS肺楔形切除28例；2例转移癌和2例小细胞肺癌（small cell lung cancer, SCLC）采用VATS肺楔形切除术。其中21个肺微小结节于术前行计算机断层扫描（computer tomography, CT）引导下Hook-wire定位，无明显并发症；其余均为手指触诊或者依据CT影像及解剖位置定位，均成功。

### 2.2 病理结果

共129个微小结节，实性结节（solid pulmonary nodule, SPN）37个，恶性比例是24.3%（9/37），术后病理结果为：肺原发性鳞状细胞癌3个，浸润性腺癌（invasive adenocarcioma, IA）3个，转移癌2个，SCLC 1个，错构瘤16个，其他炎症等良性病变12个（表 1）。49个mGGO的恶性比例是63.3%（31/49），术后病理结果为：IA 19个，微浸润腺癌（micro invasive adenocarcioma, MIA）6个，原位腺癌（adenocarcioma *in situ*, AIS）4个，非典型性腺瘤样增生（atipical adenomatous hyperplasia, AAH）1个，SCLC 1个，炎症等良性病变18个（表 2）。43个pGGO的恶性比例是86.0%（37/43），术后病理结果为：AIS 19个，MIA 6个，IA 6个，AAH 6个，炎症等良性病变6个（表 3）。GGO总的恶性比例是73.9%（68/92）。另有6例患者术中楔形切除后快速冰冻病理存在误差，术中冰冻为AAH，术后为AIS、MIA或者IA，其中2例年轻患者（AIS和IA）选择二次手术行肺叶切除和淋巴结清扫，其余患者（3个AIS和1个MIA），选择保守观察（表 4）。

**1:** 37个SPN病理类型分析 Pathological analysis of 37 SPN

Pathological analysis	*n*	Percent (%)
Hamartoma	16	43.2
Lymphonodus	3	8.1
Squmaous cell lung cancer	3	8.1
IA	3	8.1
Inflammatory granuloma	3	8.1
Metastatic carcinoma	2	5.4
Inflammation	2	5.4
Hyaline degeneration	1	2.7
Pulmonary sclerosing hemangioma	1	2.7
Inflammatory pseudotumor	1	2.7
SCLC	1	2.7
Scar	1	2.7
SPN: solid pulmonary nodule; IA: invasive adenocarcioma; SCLC: small cell lung cancer.		

**2:** 49个mGGO病理类型分析 Pathological analysis of 49 mGGO

Pathological analysis	*n*	Percent (%)
IA	19	38.7
MIA	6	31.6
AIS	4	8.2
AAH	1	2.0
SCLC	1	2.0
Inflammatory granuloma	2	4.1
Inflammation	5	10.2
Inflammatory mucle cell tumor	1	2.0
Pulmonary sclerosing hemangioma	1	2.0
Infection of streptococcus	1	2.0
Other benign lesion	8	16.3
MIA: microinvasive adenocarcioma; AIS: adenocarcioma *in situ*; AAH: atypical adenomatoid hyperplasia.		

**3:** 43个pGGO病理类型分析 Pathological analysis of 43 pGGO

Pathological analysis	*n*	Percent (%)
AIS	19	44.2
MIA	6	14.0
AAH	6	14.0
IA	6	14.0
Inflammation	4	9.3
Pulmonary sclerosing hemangioma	1	2.3
Other benign lesion	1	2.3

**4:** 冰冻病理误差 Frozen pathological error

No.	Frozen pathology	Postoperative pathology	Management
1	AAH	IA	Reoperation
2	Sclerosing hemangioma	AIS	Reoperation
3	AAH	AIS	Follow up
4	AAH	AIS	Follow up
5	AAH	AIS	Follow up
6	AAH	MIA	Follow up

NSCLC共73例，其中有淋巴结病理结果的45例中，只有2例以SPN为表现的IA出现纵隔淋巴结转移，其余均未出现淋巴结转移。术后随访1个月-35个月，平均（15.1±10.2）个月，无复发及转移。

## 3 讨论

按国际肺癌研究协会/美国胸科学会/欧洲呼吸学会对肺腺癌进行的新的病理分类^[5]^，肺部微小结节可以为浸润前病变，包括AAH、AIS，亦可以是浸润性病变，包括MIA、IA^[6]^。微小SPN以良性病变如错构瘤和淋巴结居多，在影像学上多表现为边界清楚的、近胸膜或叶裂的小球形病灶，本组恶性比例是24.3%（9/37），其中3例鳞癌均表现为SPN。恶性SPN在影像学上多有恶性征象，如分叶、毛刺、血管集束征等，较容易鉴别。而GGO在CT上表现为肺的局部密度轻度增高，呈边界模糊或清楚的磨玻璃影，其内可以观察到血管及支气管影。根据其内是否含有实性成分将其分pGGO和mGGO。pGGO是病变组织沿肺泡壁伏壁生长，不伴有肺泡结构的破坏，比如炎症、局灶性肺出血、AAH、AIS等；当病理组织增多，逐步演变为含实性成分的mGGO，如MIA、IA、肺间质纤维化等^[7, 8]^。对GGO及病理之间的关系研究很多^[9-11]^，GGO尤其是mGGO具有较高的恶性率，但外科处理后预后良好。有研究^[12]^显示持续存在的稳定的pGGO恶性概率高达59%。国内文献报道^[13]^肺部≤10 mm的GGO的恶性比例高达68%。本组mGGO的恶性比例是63.3%（31/49），pGGO的恶性比例是86.0%（37/43），GGO总的恶性比例是73.9%（68/92）。总体而言，与SPN相比，GGO与肺癌关系更为密切，尤其是mGGO，常常高度提示肺腺癌^[14, 15]^。除少数个案报道提出GGO最终被确诊为鳞癌，其余研究^[16, 17]^均证实GGO与肺鳞癌无直接相关性癌。因此肺微小GGO应予以积极处理，对肺癌的早期诊断和治疗、降低死亡率具有重要意义。但目前对于肺微小结节无明确诊断及手术方案^[18]^。由于部分容积效应的影响，正电子发射计算机断层显像（positron emission tomography-CT, PET-CT）对于肺微小结节的鉴别意义不大^[19, 20]^。近年出现早期NSCLC行部分肺叶切除和选择性淋巴结切除^[21, 22]^。直径＜2 cm的早期肺癌肺段切除和肺叶切除的远期生存率无差异^[23]^。Miller等^[24]^对≤10 mm肺癌行肺叶切除、亚肺叶切除（肺段切除和楔形切除）进行对比，生存率和局部复发率没有统计学差异。病理类型为AIS、MIA的pGGO的手术方式已推荐为亚肺叶切除（肺段或楔形切除）而非肺叶切除^[25]^。VATS楔形切除技术简单、并发症少及死亡率低，主要用于高龄、合并心肺功能不全^[26]^的微小肺癌患者。系统性淋巴结清扫并未给早期肺癌患者带来更好的生存获益^[27]^。研究^[28-30]^显示GGO所占比例≥50%的早期NSCLC均未出现淋巴结转移，可不行系统性淋巴结切除。本组中有淋巴结病理的mGGO和pGGO，均未出现淋巴结转移，2例SPN出现淋巴结转移，病理类型为浸润性腺癌。mGGO的恶性程度与实性成分所占比例有关，实性成分的增多提示着恶性病变存在进展可能^[31]^。mGGO实性成分增多或者SPN应考虑行选择性或系统性淋巴结切除^[30]^。美国和日本的两项关于早期肺癌切除范围的多中心随机对照临床试验正在进行，结果一旦揭晓，将有一个最客观的定论来规范微小肺癌外科治疗的标准术式。CT引导下Hook-wire定位GGO，成功率高，并发症轻微^[32, 33]^。

术中冰冻技术日益成熟，但由于肿瘤异质性、冰冻病理的局限性及病理医师的诊断经验，术中冰冻病理存在一定误差，尤其是鉴别AAH、AIS、MIA等低度恶性肿瘤，仍存在一定的局限。术中冰冻技术对直径≤10 mm肺部微小结节的灵敏度仅为86.9%，对于11 mm-15 mm的结节灵敏度为94.1%，≤5 mm的结节不适合术中快速冰冻病理检查^[34, 35]^。术中为良性或者低度恶性，而术后为恶性或者浸润性癌的情况依然存在，二次手术行根治肺叶切除和系统性淋巴结切除的情况亦会发生，因此围手术期应与患者及家属充分沟通，避免医疗纠纷。本组中有6例书中冰冻病理出现误差，经充分沟通，患者及家属理解，其中2例接受二次手术治疗，其余均随访。

综上所述，有恶性表现、或有危险因素及持续存在的肺部微小结节尤其是GGO，是恶性病灶的概率大，应积极外科处理；围手术期应与患者及家属充分告知冰冻病理结果存在误差可能性，避免医疗纠纷。
